# Keeping it in the family: Coevolution of latrunculid sponges and their dominant bacterial symbionts

**DOI:** 10.1002/mbo3.417

**Published:** 2016-10-26

**Authors:** Gwynneth F. Matcher, Samantha C. Waterworth, Tara A. Walmsley, Tendayi Matsatsa, Shirley Parker‐Nance, Michael T. Davies‐Coleman, Rosemary A. Dorrington

**Affiliations:** ^1^ Department of Biochemistry and MicrobiologyRhodes UniversityGrahamstownSouth Africa; ^2^ Department of BiotechnologyVaal University of TechnologyVanderbijlparkSouth Africa; ^3^ Department of ChemistryRhodes UniversityGrahamstownSouth Africa; ^4^ Faculty of Natural ScienceUniversity of the Western CapeCape TownSouth Africa

**Keywords:** Betaproteobacterium, Latrunculiidae, Spirochaetae, symbionts

## Abstract

The Latrunculiidae are a family of cold water sponges known for their production of bioactive pyrroloiminoquinone alkaloids. Previously it was shown that the bacterial community associated with a *Tsitsikamma* sponge species comprises unusual bacterial taxa and is dominated by a novel Betaproteobacterium. Here, we have characterized the bacterial communities associated with six latrunculid species representing three genera (*Tsitsikamma*,* Cyclacanthia*, and *Latrunculia*) as well as a *Mycale* species, collected from Algoa Bay on the South African southeast coast. The bacterial communities of all seven sponge species were dominated by a single Betaproteobacterium operational taxonomic unit (OTU_0.03_), while a second OTU_0.03_ was dominant in the *Mycale* sp. The Betaproteobacteria OTUs from the different latrunculid sponges are closely related and their phylogenetic relationship follows that of their hosts. We propose that the latrunculid Betaproteobacteria OTUs are members of a specialized group of sponge symbionts that may have coevolved with their hosts. A single dominant Spirochaetae OTU_0.03_ was present in the *Tsitsikamma* and *Cyclacanthia* sponge species, but absent from the *Latrunculia* and *Mycale* sponges. This study sheds new light on the interactions between latrunculid sponges and their bacterial communities and may point to the potential involvement of dominant symbionts in the biosynthesis of the bioactive secondary metabolites.

## 1 Introduction

The first sponges (phylum Porifera) emerged more than 700 million years ago and their modern day descendants represent some of the earliest metazoans (Brain et al., 2012; Maloof et al., 2010). Marine sponges are filter‐feeders that lack differentiation and specialized organs, acquiring nutrients through phagocytosis of particulate matter consisting mainly of microorganisms from the surrounding water (Simpson, 1984). Sponges have evolved close associations with bacteria, which are found as extracellular symbionts in the mesohyl matrix or intracellular symbionts (Burgsdorf et al., 2015; de Voogd, Cleary, Polónia, & Gomes, 2015; Hentschel, Piel, Degnan, & Taylor, 2012; Moitinho‐Silva et al., 2014). Sponge–bacterial symbionts can account for up to 40% of the mass of the animal at densities several orders of magnitude higher than in the surrounding seawater (Hentschel, Usher, & Taylor, 2006; Hoffmann et al., 2005). These microbial communities provide services that are essential for sponge fitness and are involved in carbon, nitrogen, and sulfur nutrient cycling (de Voogd et al., 2015; Hentschel et al., 2006; Ribes et al., 2012). In addition, the symbiotic bacteria produce secondary metabolites that are postulated to be used by the host as a chemical defense against predation, fouling, and diseases (Freeman, Vagstad, & Piel, 2016; Hentschel et al., 2006, 2012; Keren, Lavy, Mayzel, & Ilan, 2015; Taylor, Radax, Steger, & Wagner, 2007; Wakimoto et al., 2014).

Sponge‐associated bacterial communities are distinct from those in the surrounding seawater (de Voogd et al., 2015; Moitinho‐Silva et al., 2014; Taylor et al., 2007) representing more than 41 different phyla and forming sponge‐specific phylogenetic clusters (Lafi et al., 2009; Simister, Deines, Botte, Webster, & Taylor, 2012; Taylor et al., 2007; Thomas et al., 2016; Webster et al., 2010). Early characterization of rRNA gene sequences provided evidence supporting the notion of a sponge‐specific microbiome distinct from the surrounding seawater (Hentschel et al., 2002; Taylor et al., 2007; Hentschel et al., 2012). However, analysis of deep sequence datasets has revealed that more than half of previously considered sponge‐specific taxa are also found in marine sediments and seawater albeit at significantly lower abundances (Taylor et al., 2013; Webster et al., 2010). In general, the conservation of sponge‐associated microbial communities does not follow host lineages but rather appears to relate to geographical distribution (Erwin, López‐Legentil, González‐Pech, & Turon, 2012; Erwin, Olson, & Thacker, 2011; Hardoim et al., 2012; Schmitt et al., 2012). The majority of bacterial symbionts are not host‐specific (Alex, Silva, Vasconcelos, & Antunes, 2013; Fiore, Jarett, & Lesser, 2013; Schmitt et al., 2012), supporting the hypothesis that the sponge‐associated bacterial communities are recruited from their habitat (Alex & Antunes, 2015; Erwin et al., 2012).

The Latrunculiidae (Demospongiae, Poecilosclerida) are a family of cold water sponges occurring predominantly in the Southern Hemisphere in rocky reef habitats up to 50 m in depth in the subtidal zone. The family comprises five genera, four of which (*Cyclacanthia*,* Latrunculia*,* Strongylodesma*, and *Tsitsikamma*) include species that are endemic to the south‐eastern coast of South Africa (van Soest, 2015). Latrunculid sponges are known for their production of bioactive pyrroloiminoquinone alkaloids such as the makaluvamines, tsitsikammamines, and discorhabdins (Antunes, Copp, Davies‐Coleman, & Samaai, 2005) that have potential for anticancer and antimalarial drug development (Antunes et al., 2004, 2005; Davis et al., 2012). Pyrroloiminoquinones are not only found in latrunculid sponges, but have also been isolated from species belonging to other families as well as ascidians, myxomycetes, and actinomycetes (Copp, Ireland, & Barrows, 2002; Davis et al., 2012; Hughes, MacMillan, Gaudêncio, Jensen, & Fenical, 2009; Ishibashi et al., 2001; Miyanaga et al., 2011; Nagata et al., 1997) leading to the suggestion that the biosynthetic origin of latrunculid pyrroloiminoquinones is likely to be microbial.

A previous study showed that bacterial communities associated with a *Tsitsikamma* sponge species are dominated by a unique Betaproteobacterium operational taxonomic unit (OTU_0.03_). Sequence reads assigned to phyla other than the Proteobacteria did not cluster with sponge‐associated sequences, suggesting that this *Tsitsikamma* species might host a unique microbial community (Walmsley et al., 2012). In this study, we set out to determine whether Betaproteobacteria are the dominant bacterial taxa in other, related latrunculid sponge species particularly in those species (*Tsitsikamma* and *Cyclacanthia*) which produce pyrroloiminoquinones as secondary metabolites.

## 2 Materials and Methods

### 2.1 Sampling and taxonomic identification of sponge specimens

All sponge samples were collected between April 2009 and August 2014 by SCUBA at Evans Peak and Riy Banks in Algoa Bay, South Africa at depths of 25–30 m (Table 1). The sponges were morphologically identified (S. Parker‐Nance and R. A. Dorrington, unpublished data) and voucher samples have been deposited at the South African Institute for Aquatic Biodiversity (SAIAB), Grahamstown, South Africa. Sponge specimens were collected in Ziploc bags and seawater and sediment were collected from their immediate vicinity concurrent with the sampling of *Tsitsikamma favus* TIC2014‐001. These were kept at 4°C until processing back at the laboratory (within 3 hr of collection). In order to remove any transient surface bacteria, the sponges were rinsed with sterile artificial seawater and then processed as described in Walmsley et al. (2012). Sediment and seawater samples were collected along with sponge TIC2014‐001 at Evans Peak. The sediment was preserved in RNA‐*Later* ^™^ and stored at −20°C until further processing. Marine bacteria were collected by filtering 2 L seawater through a 0.2‐μm polyethersulfone (PES) filter (Pall), which was preserved in RNA‐Later and stored at −20°C. The taxonomic identities of sponges were confirmed by the analysis of 28S rRNA gene sequence amplified from gDNA using the primer pairs RD3a (5′‐GAC CCG TCT TGA AAC ACG A‐3′) and RD5b2 (5‐ACA CAC TCC TTA GCG GA‐3′) producing a 625 nucleotide amplicon. Resultant gene sequences were submitted to GenBank with the accession numbers KU535622, KC471505, KC471509, KC471503, KU535626, KU535627, KC471509, KU535629, and KC471506.

**Table 1:** Taxonomic identification and collection data for sponge specimens used in this study

Collection number	Taxonomicidentification	Collection site	Collection date	Depth	Nucleic acid extraction method	454 Sequence reads analyzed
TIC2009‐002^a^	*Tsitsikamma favus*	Evans Peak33°50.578S; 25°48.988E	May 2009	30 m	Guanidine thiocyanate(Walmsley et al., 2012)	891
TIC2010‐070	*Tsitsikamma* sp. 005	Evans Peak33°50.578S; 25°48.988E	May 2010	30 m	Guanidine thiocyanate(Walmsley et al., 2012)	14,070
TIC2010‐2B	*Tsitsikamma* sp. 004	Evan's Peak33°50.578S; 25°48.988E	May 2010	30 m	Guanidine thiocyanate(Walmsley et al., 2012)	4,497
TIC2010‐031^b^	*Latrunculia algoaensis*	Evans Peak33°50.578S; 25°48.988E	May 2010	30 m	Guanidine thiocyanate(Walmsley et al., 2012)	10,460
TIC2010‐030	*Mycale (Mycale)* sp. 001	Evans Peak33°50.578S; 25°48.988E	May 2010	30 m	Guanidine thiocyanate(Walmsley et al., 2012)	22,625
TIC2011‐102	*Tsitsikamma* sp. 002	Evans Peak33°50.578S; 25°48.988E	April 2011	30 m	Guanidine thiocyanate(Walmsley et al., 2012)	11,217
TIC2012‐057	*Tsitsikamma favus*	Evans Peak33°50.578S; 25°48.988E	Dec 2012	30 m	ZR Bacterial Miniprep kit AllPrep DNA/RNA MiniKit (Qiagen)	3,2602,960 (gDNA)2,918 (cDNA)
TIC2012‐056	*Cyclacanthia bellae*	Riy Banks33°59.960S; 25°58.764E	Dec 2012	25–30 m	ZR Bacterial Miniprep kit AllPrep DNA/RNA MiniKit (Qiagen)	2,9592,061(gDNA)2,040 (cDNA)
TIC2014‐001	*Tsitsikamma favus*	Evans Peak33°50.578S; 25°48.988E	Aug 2014	30 m	ZR Bacterial Miniprep kit (Zymo: D6005)	17,706
Sediment	NA	Evans Peak33°50.578S; 25°48.988E	Aug 2014	39 m	ZR Soil Microbe DNA MiniPrep Kit (Zymo D6001)	3,281
Seawater	NA	Evans Peak33°50.578S; 25°48.988E	Aug 2014	30 m	MoBio PowerWater DNA Isolation kit (MoBio, 14900)	2,218

a Previously identified as *Tsitsikamma favus* by Walmsley et al. (2012).

b Morphological description published in Samaai et al. (2012). Collection details in Samaai et al. (2012) are incorrect.

### 2.2 Genomic DNA extraction and 16S rRNA gene amplification

Sponge samples were homogenized using a pestle and mortar with 3 ml of artificial seawater. The homogenized tissue was then transferred to 1.5 ml Eppendorf tubes and centrifuged at 15,000*g* for 1 min. Total genomic DNA/RNA was extracted from the resulting pellet as indicated in Table 1. Amplicon libraries of the hypervariable regions 4 and 5 of the 16S rRNA gene were created using the primer pair E517F (5′‐CAG CAG CCG CGG TAA‐3′) and E969‐983 (5′‐GTA AGG TTC YTC GCG T‐3′) (Wang, Garrity, Tiedje, & Cole, 2007; Wang & Qian, 2009) with relevant multiplex identifier (MID) tags. Amplification was done using KAPA HiFi HotStart Taq (KAPA Biosystems) in a reaction volume of 25 μl as per the manufacturer's specifications. Nested PCR was done for sponges collected prior to 2011 as described in Matcher, Dorrington, Henninger, and Froneman (2011). For sponges collected in 2012 and 2014, PCR of the template genomic DNA using MID‐tagged primers was done. In this instance, cycling parameters were as follows: initial denaturation and enzyme activation at 98°C for 5 min, 5 cycles of 98°C for 45 s, 45°C for 30 s, 72°C for 45 s, followed by 18 cycles of 98°C for 30 s, 50°C for 30 s, 72°C for 45 s. A final extension was done at 72°C for 5 min. The resulting amplicon products were purified using Agencourt AMPure XP beads (Beckman Coulter). Amplicon libraries were sequenced using the GS Junior Titanium Sequencing platform (454 Life Sciences, Roche) as per the manufacturer's specifications.

### 2.3 Data curation and analyses

Curation of the sequence datasets was done using the Mothur platform (Schloss et al., 2009) in which low‐quality reads, including reads shorter than 250 bp in length, any reads with ambiguous nucleotides, or reads in which homopolymeric runs greater than 7 were observed, were removed. Chimeras were identified using the UCHIME algorithm (Edgar, Haas, Clemente, Quince, & Knight, 2011) and then removed. Classification of the sequence reads was done using Naïve Bayesian classifier against the Silva bacterial database (release version 119) and then plotted as a percentage of the total number of reads per sample. A distance matrix (cut‐off of 0.15) was generated in Mothur and used to cluster the sequence reads into operational taxonomic units at distance values of 0.03, 0.01, and unique (i.e., distance value of zero). Betaproteobacterial and Spirochaetae sequences from sponges reported in the literature were obtained from the NCBI nucleotide database. Phylogenetic trees were constructed with the MEGA6.06 software (Tamura, Stecher, Peterso, Filipski, & Kunar, 2013) using ClustalW and Neighbor‐joining algorithms.

The sequence datasets generated in this study have been deposited in the sequence reads archive (SRA) database of the National Centre of Biotechnology Information (SRA accession: SRP073045).

## 3 Results

### 3.1 Sponge collection, diversity, and phylogeny

Nine sponge specimens, representing three endemic genera of the family Latrunculiidae, were collected over a period of 6 years from two shallow water rocky reef sites in Algoa Bay (Table 1). Taxonomic identification revealed three new *Tsitsikamma* species: *Tsitsikamma favus* including one specimen each of *Tsitsikamma* sp. 002, *Tsitsikamma* sp. 004, and *Tsitsikamma* sp. 005. The new *Tsitsikamma* species have been morphologically described and their phylogenetic relationship with other members of the genus and within the Latrunculiidae family confirmed. The *Mycale* sp. 001 was found growing as an encrusting sponge on the *Tsitsikamma* sp. 005 (TIC2010‐070) specimen as well as on other specimens of *Tsitsikamma* sp. 002, not included in this study. The taxonomic identity of sponges was confirmed by the analysis of partial 28S rRNA gene sequence.

### 3.2 Sponge‐associated bacterial communities

**Figure 1:**
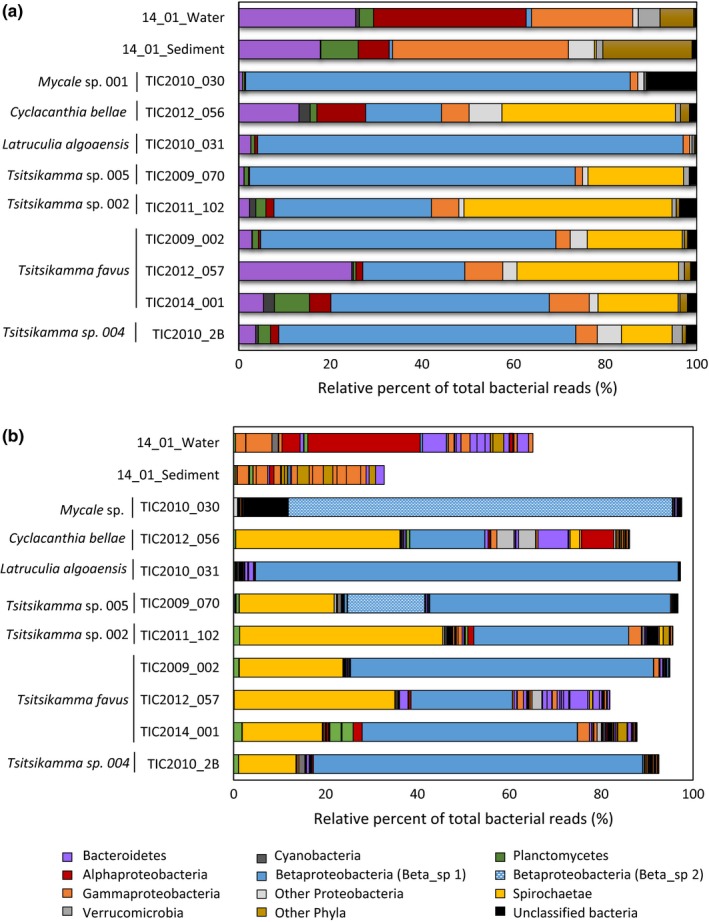
Phylogenetic classification of sponge–bacterial communities and identification of dominant bacterial species. (a) Phylogenetic classification (at the level of phylum and subphylum) of the bacterial communities in sponges, sediment, and seawater using Naïve Bayesian classification with the Silva bacterial database (version 119) as the reference. (b) Classification of the top 20 dominant OTUs (at a distance of 0.03) in each of the samples. Read abundance is indicated as the relative percentage of the total bacterial reads analyzed for each sponge

A total of 103,163 sequence reads, spanning the V4–V5 regions of the bacterial 16S rRNA gene, were used for phylogenetic classification of the bacterial taxa associated with the sponges and in the sediment and seawater. There was a striking difference between sponge‐associated bacterial communities and those in the surrounding seawater and sediment. Betaproteobacteria were dominant in the sponge communities, accounting for as much as 84% and 93% of the sequence reads obtained for the *Mycale* sp. 001 and *Latrunculia algoaensis*, respectively (Figure 1a). Betaproteobacteria also dominated the *Tsitsikamma* bacterial assemblages, although Spirochaetae were also found in high abundance with relative percentages ranging from 11% to 45% of the total reads in these sponges. By contrast, the dominant phyla in the environment were Gammaproteobacteria (38% and 22% in seawater and sediment, respectively) as well as Alphaproteobacteria, which represented more than 33% of the reads in the seawater (Figure 1a). There were also significant numbers of reads classified as Bacteroidetes in the water and sediment with taxa belonging to this phylum also present in all the sponges.

Overall, the bacterial species richness was significantly higher in the environment (seawater and sediment) compared to the sponges (Figure 1b). The numerically dominant Betaproteobacteria in the latrunculid sponges were represented by the same OTU_(0.03)_ (B_OTU1). This OTU was found at low levels (<0.05% of reads) in the seawater and was absent in the sediment. In the *Mycale* sp. sponge, the Betaproteobacteria were dominated by a different OTU_(0.03)_ (B_OTU2) with 95% sequence identify to B_OTU1. B_OTU2 was not only absent in the seawater and sediment, but also present in the *Tsitsikamma* sp. 005 specimen on which the *Mycale* sp. was found growing as an encrusting sponge. The Spirochaetae were represented by a single OTU_(0.03)_, which were neither found in the *L. algoaensis* and *Mycale* sp. nor in the seawater and sediment. While no dominance was observed for any single OTU in the sediment, an Alphaproteobacterial OTU_(0.03)_ (identified as *Pelagibacter* species) was dominant in the seawater, which is to be expected as *Pelagibacter* are prevalent in aquatic ecosystems (Giovannoni & Vergin, 2012). This OTU was present in all the sponge samples, where it accounted for between 0.03% and 1.9% of the total bacterial reads (Figure 1b).

### 3.3 Phylogeny of the sponge‐associated Betaproteobacteria

**Figure 2:**
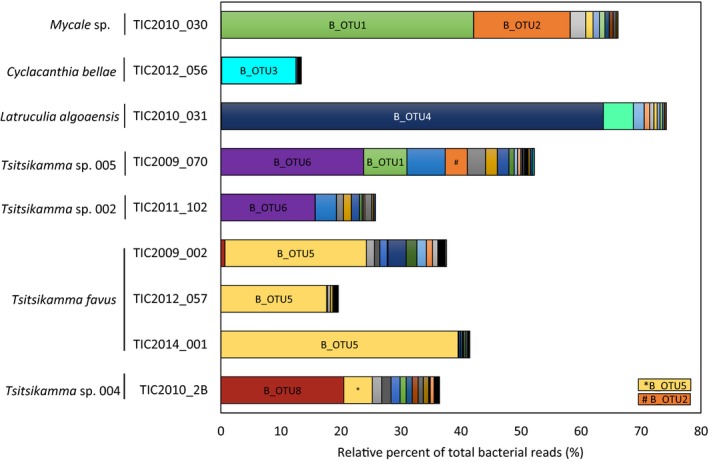
Dominant, unique (0.00) Betaproteobacteria OTUs. Read abundance is indicated as the relative percentage of the total bacterial reads analyzed for each sponge

With the exception of *Tsitsikamma* sp. 005, the dominant betaproteobacterial OTU in each sponge was represented by a single lineage (Figure 2). Based on the 16S rRNA gene sequence, the *T. favus* sponges, which were collected over a period of 6 years from the same location, all harbor the same unique OTU (B_OTU5), which was also present in the closely related *Tsitsikamma* sp. 004 sponge, where B_OTU8 was dominant. The dominant Betaproteobacterium in *Tsitsikamma* sp. 002 and *Tsitsikamma* sp. 005 was B_OTU6, while the *C*. *bellae* and *L*. *algoaensis* sponges each contained a unique OTU_(0.00)_ (B_OTU3 and B_OTU4, respectively) not found in the *Tsitsikamma* sponges. Finally, B_OTU1 and B_OTU2, which were associated with the *Mycale* sp. 001 were also found in the *Tsitsikamma* sp. 005 sponge. Since the *Mycale* sp. was found as an encrusting sponge on this specimen, the presence of these two OTUs is likely due to cross‐contamination during removal of the encrusting *Mycale* tissue (Figure 2).

**Figure 3:**
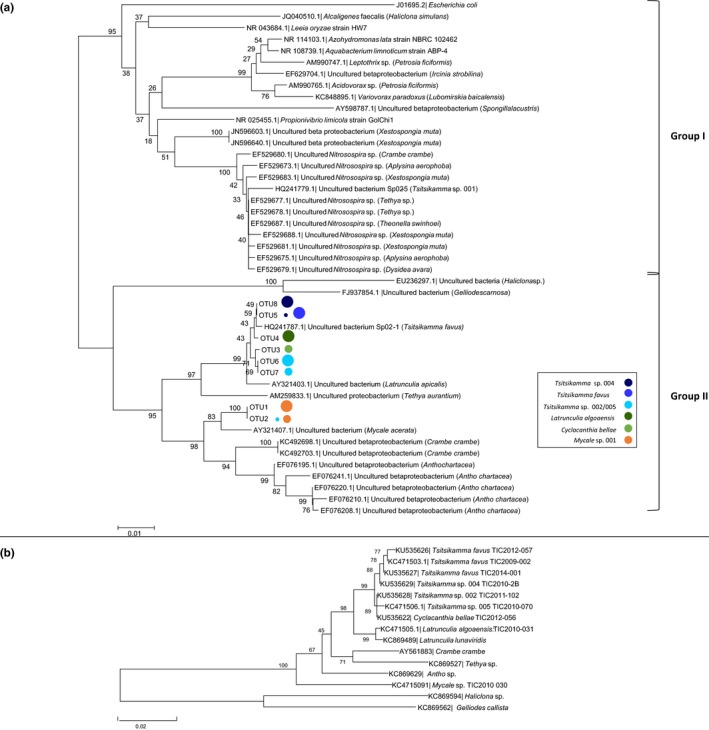
Phylogenetic relationship between dominant Betaproteobacteria OTUs and other sponge‐associated Betaproteobacteria and their sponge hosts. (a) Phylogenetic analysis of sponge‐associated Betaproteobacteria OTUs. Related Betaproteobacteria sequences were obtained from GenBank (corresponding accession number attached) and, where possible, the sponge host is provided. The phylogenetic analysis was generated using MEGA6 software (Tamura et al., 2013) with the Neighbor‐joining method (Saitou & Nei, 1987). The 16S rRNA gene sequence of *Escherichia coli* (J01695) was used to root the tree. Bootstrap values, calculated based on 1,000 replicates, are indicated next to the branches (Felsenstein, 1985). Evolutionary distance was calculated using the maximum composite likelihood method (Tamura, Nei, & Kumar, 2004) based on the number of nucleotide substitutions per site over a total of 408 positions. The host of each OTU is indicated by a color‐coded circle with large versus small circles indicating relative dominance. (b) Phylogeny of sponges harboring Betaproteobacteria that cluster in Group II based on partial 28S rRNA gene sequences. The phylogenetic analysis was generated using MEGA6 software as described above. Evolutionary distance was calculated using the maximum composite likelihood method based on the number of nucleotide substitutions per site over a total of 625 positions. Accession numbers are indicated in brackets

**Figure 4:**
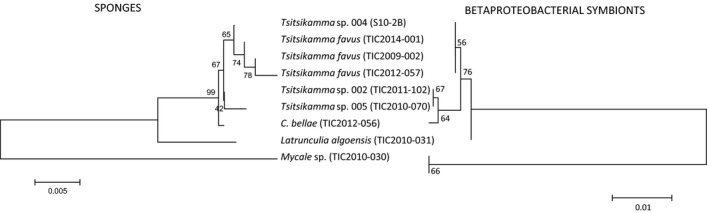
Sponge host versus Betaproteobacterial symbiont phylogenies generated using MEGA6 software and the Neighbor‐joining method with 5,000 replicates (bootstrap values are shown next to the branches). The trees are drawn to scale with the branch length units the same as those of the evolutionary distance. The evolutionary distances were calculated using the maximum composite likelihood method. Congruent topologies were also generated when using maximum likelihood and maximum parsimony methods

An analysis of the phylogenetic relationship between the dominant betaproteobacterial OTUs and other sponge‐associated Betaproteobacteria shows the presence of two large monophyletic clusters (Figure 3a). The first (Group I) comprises both sponge‐associated and free‐living bacterial species, including sponge‐associated members of the ammonia‐oxidizing genus, *Nitrosospira*. Clustering within this genus is clone Sp02‐5, representing a Betaproteobacterium OTU from a 16S rRNA clone library generated in an earlier study on *T. favus* TIC2009‐002 (Walmsley et al., 2012). The second group (Group II) comprises uncultured sponge‐associated taxa and includes all the betaproteobacterial OTUs identified in this study (Figure 3a). The latrunculid OTUs form a cluster that includes the 16S rRNA sequence Sp02‐1, representing the dominant OTU in a clone library derived from *T. favus* TIC2009‐002 generated in a previous study (Walmsley et al., 2012) and an uncultured proteobacterium from *Latrunculia apicalis* (Figure 3a). B_OTU8 and B_OTU5, which were found in *Tsitsikamma* sp. 004 and *T. favus*, respectively, are closely related with greater than 99% identify and form a cluster with B_OTU4 from *L*. *algoensis*. Similarly, B_OTU6 and B_OTU7, which dominate in *Tsitsikamma* sp. 002 and *Tsitsikamma* sp. 005 are also very closely related clustering together with B_OTU3, which is the dominant OTU in *C*. *bellae*. The closest relative to the latrunculid clade is a *Tethya aurantium*‐associated Betaproteobacterium. The dominant betaproteobacterial OTUs from the *Mycale* sp. were found most closely related to an uncultured betaproteobacterial sequence from *Mycale acerata* and in a separate clade with Betaproteobacteria associated with *Crambe crambe* and *Antho chartacea* (Figure 3a). Strikingly, the phylogenetic relationship of these betaproteobacterial OTUs closely resembles that of their host sponges (Figure 4) suggesting coevolution of these sponges and their associated Betaproteobacteria.

### 3.4 Sponge‐associated Spirochaetae phylogenies

**Figure 5:**
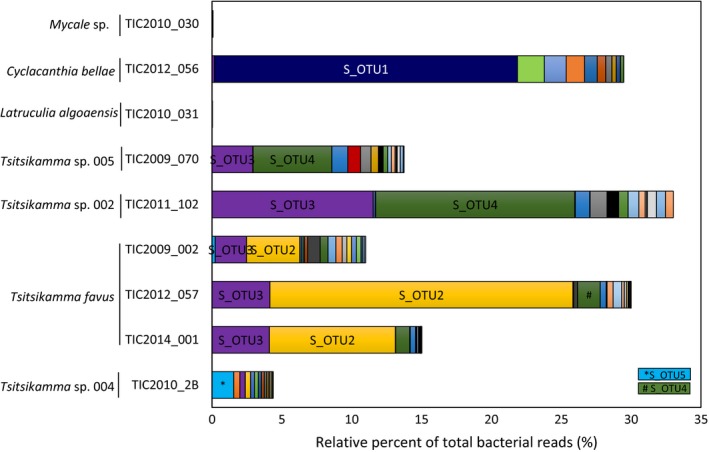
Dominant unique (0.00) Spirochaetae OTUs from each of the sponges. Read abundance is indicated as the relative percentage of the total bacterial reads analyzed for each sponge

While the phylum Spirochaetae was not represented in the *Mycale* sp. and *L*.* algoaensis* sponges, bacteria belonging to this phylum formed a significant proportion of the bacterial population within sponges from the genus *Tsitsikamma* and in the *C*. *bellae* specimen (Figure 1a). As observed for the Betaproteobacteria, the Spirochaetae reads were also dominated by a single OTU_(0.03)_ (Figure 1b) and further analysis revealed the same striking dominance of unique OTUs as observed for the Betaproteobacteria. A single, Spirochaetae lineage, S_OTU1, was dominant in *C. bellae*, accounting for over 21% of the total sequence reads for this sample (Figure 5). S_OTU1 was not found in any of the *Tsitsikamma* sponges. There were four unique *Tsitsikamma*‐specific Spirochaetae OTUs, the distribution and relative abundances of which appear to be species related (Figure 5). S_OTU2 and S_OTU4 were present, but not dominant in all the *Tsitsikamma* sponges, with the dominance of S_OTU4 being confined to *Tsitsikamma* sp. 002 and *Tsitsikamma* sp. 005. The dominant spirochete in all three *T. favus* specimens, S_OTU2, also occurred in the closely related *Tsitsikamma* sp. 004 (Figure 3b), but was absent in *Tsitsikamma* sp. 002 and *Tsitsikamma* sp.005. While S_OTU5 was dominant, *Tsitsikamma* sp. 004 appeared to have fewer Spirochaetae within its symbiotic bacterial community as compared to other *Tsitsikamma* species. Since we were only able to collect a single specimen of this species, it is unclear whether this observation is true for the species as a whole.

**Figure 6:**
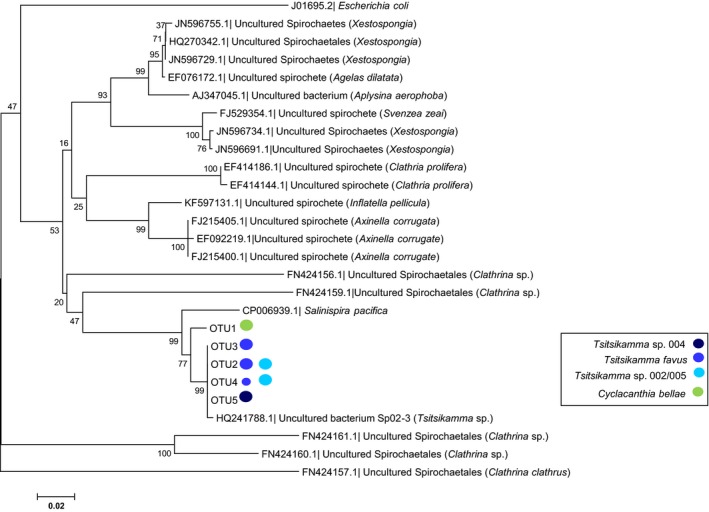
Phylogenetic relationship between dominant latrunculid Spirochaetae OTUs and other predominantly sponge‐associated Spirochaetes. Related Spirochaetae sequences were obtained from GenBank (corresponding accession number indicated) and, where possible, the sponge host is provided. The phylogenetic analysis was generated using MEGA6 software (Tamura et al., 2013) with the Neighbor‐joining method (Saitou & Nei, 1978). The 16S rRNA gene sequence of *Escherichia coli* (J01695) was used to root the tree. Bootstrap values, calculated based on 1,000 replicates, are indicated next to the branches (Felsenstein, 1985). Evolutionary distance was calculated using the maximum composite likelihood method (Tamura et al., 2004) based on the number of nucleotide substitutions per site over a total of 259 positions. The branch lengths correlate with evolutionary distance used to infer the phylogenetic tree. The host of each OTU is indicated by a color‐coded circle with large versus small circles indicating relative dominance

Phylogenetic analysis revealed that the *Tsitsikamma* Spirochaetae OTUs are extremely closely related, clustering with the dominant Spirochaetae 16S rRNA gene sequence (Sp02‐3) obtained from a clone library of *T. favus* (Walmsley et al., 2012) (Figure 6). S_OTU1, the dominant OTU from *C*. *bellae*, is less closely related to the *Tsitsikamma* OTUs. A BLAST analysis showed that the closest relative to the Sp02‐3 16S rRNA sequence, with 93% identify, is a strain of *Salinispira pacifica* that was isolated from hypersaline microbial mats (Ben Hania et al., 2015). With respect to sponge‐associated spirochaetes, the nearest phylogenetic relative (<90% sequence identity to the latrunculid Spirochaetae OTUs) was associated with sponges belonging to the genus *Clathrina* (Figure 6). Thus, the *Tsitsikamma* and *Cyclacanthia* Spirochaetae OTUs represent members of a new family of specialized sponge–bacterial symbionts.

### 3.5 Metabolically active bacterial symbionts

**Figure 7:**
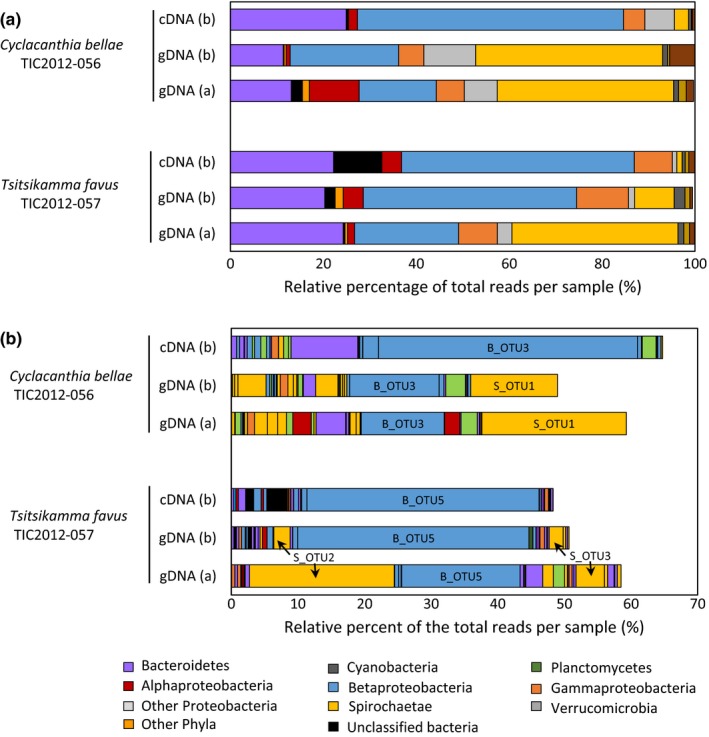
Identification of metabolically active bacterial species in *T. favus* and *C. bellae*. (a) Phylogenetic classification (at the level of phylum and subphylum) of the 16S rRNA gene sequence (gDNA) versus 16S rRNA sequences (cDNA) of bacterial communities in *T. favus* (TIC2012‐057) and *C. bellae* (TIC2012‐056). Classification was conducted using Naïve Bayesian classification with the Silva bacterial database (version 119) as the reference. Genomic DNA was extracted from two individual segments of each sponge using two different extraction protocols as technical replicates (i.e., gDNA “a” and gDNA “b”). gDNA (a) was extracted using the ZR Bacterial Miniprep kit (Zymo), while gDNA (b) was extracted from the same tissue segment using the AllPrep DNA/RNA MiniKit (Qiagen). (b) Classification of top 20 dominant OTUs (at a distance of 0.00) from each of the samples. Read abundance is indicated as the relative percentage of the total bacterial reads analyzed for each sponge

To better understand the interaction between the latrunculid sponges and their dominant Betaproteobacteria and Spirochaetae OTUs, we focused on identifying the metabolically active bacterial taxa in *T. favus* (TIC2012‐057) and *C. bellae* (TIC2012_056). An extraction protocol that allowed for simultaneous isolation of RNA and DNA from the same sponge sample was used to allow direct correlation between numerical abundance (16S rRNA gene sequences) and 16S rRNA gene expression (16S rRNA) as a proxy for metabolically active bacterial taxa (Figure 7, “b” samples). The data generated from gDNA isolated using a different protocol (Figure 7, “a” samples) and used for earlier analyses in this study were included as an experimental control for potential bias introduced by the different nucleic acid extraction protocols. The data reveal that the Betaproteobacteria are numerically dominant and metabolically active in both latrunculid sponge species, while the Spirochaetae are numerically dominant, but do not appear to be as metabolically active (Figure 7a). An OTU_(0.00)_ analysis showed that B_OTU5 and B_OTU3 were metabolically active in *T. favus* and *C. bellae* (Figure 7b). As observed previously, the Spirochaetae OTUs, S_OTU2 and S_OTU3, were present in *T. favus*, although they were less abundant in the “b” samples (Figure 7b). In *C. bellae*, the dominant Spirochaetae OTU was S_OTU1, which occurred at similar levels in both the “a” and “b” samples, indicating that the discrepancies observed for *T. favus* were likely due to the tissue selected and not a function of the nucleic acid extraction method used.

A bacterial strain belonging to the phylum Bacteroidetes was very active in *C. bellae* (Figure 7b). BLAST analysis returned a Bacteroidetes sequence with only 87% identity, suggesting that this active OTU represents a novel Bacteroidetes taxon. There were four metabolically active cyanobacterial OTUS in the *T. favus* sponge, three of which belong to the genus *Synechococcus*, a well‐known sponge symbiont (Alex, Vasconcelos, Tamagnini, Santos, & Antunes, 2012; Burgsdorf et al., 2015; Gao et al., 2014; Simister et al., 2012). Two *Synechococcus* OTUs exhibited 100% identity with *Synechococcus* symbionts from the sponge *Hymeniacidon perlevis* (accession numbers JX477013 and JX477014) and one had 99% identity to a cyanobacterial symbiont from an *Axinella* sponge species (accession number KJ007982). The forth cyanobacterial OTU in *T. favus* was most similar to an uncultured Cyanobacterium isolated from seawater and coral reefs. While 16S rRNA gene sequence abundance generated from RNA are generally accepted as a proxy for metabolic activity (Campbell, Yu, Heidelberg, & Kirchman, 2011; Lanzen et al., 2011; Moitinho‐Silva et al., 2014), other factors may influence these abundance profiles. Specifically, small cells are likely to harbor fewer rRNAs relative to that of larger cells which may in turn misrepresent the smaller cells as being less active. A second factor that may influence the use of rRNA as a proxy for metabolic activity is the finding that some bacterial species maintain significant levels of rRNA even while dormant (Kamke, Taylor, & Schmitt, 2010; Morgenroth et al., 2000). It is unlikely that these factors have influenced our findings with respect to the Betaproteobacterium OTU, which is numerically dominant and also the species with high levels of rRNA. However, we cannot rule out the possibility that cell size and a smaller complement of ribosomes per cell might account for the apparently low levels of rRNA in the Spirochaetae OTU.

## 4 Discussion

In this study, we set out to investigate whether the unusual bacterial community associated with a *Tsitsikamma* species (Walmsley et al., 2012) was conserved in related sponge species within the Latrunculiidae family. The data reveal the conservation of a single, dominant Betaproteobacterium species within the microbiome of six different species representing three genera within the family Latrunculiidae. Remarkably, each sponge species hosts a unique Betaproteobacterium strain that is conserved in multiple specimens of the same species *T. favus* collected over a period of 6 years. A closely related betaproteobacterium has also been isolated from *Tethya* sponges. Furthermore, not only is this bacterium dominant in the sponges but is also found within the embryos of several sponge specimens (S. C. Waterworth & R. A. Dorrington, unpublished data). These data suggest that this betaproteobacterial sponge symbiont is vertically transmitted from one sponge generation to the next. The presence of a dominant Betaproteobacterium species is not restricted to latrunculid sponges collected from the same geographical location since a closely related Betaproteobacterium was reported as one of the dominant OTUs in a 16S rRNA clone library derived from *L. apicalis* collected in McMurdo Sound in the Ross Sea of Antarctica (Webster, Negri, Munro, & Battershill, 2004).

Sponges belonging to other families within the order Poecilosclerida, including the Mycalidae, Crambeidae, and Microcionidae (Fig. S1), also harbor bacterial communities dominated by Betaproteobacterial symbionts. The two dominant Betaproteobacterial OTUs from *Mycale* sp. 001 (this study) cluster with 16S rRNA clone sequences derived from the Antarctic sponge *Mycale acerata*. These clones comprise three of a total of seven obtained for *M. acerata* (Webster et al., 2004), so it is likely that this sponge species also harbors a dominant Betaproteobacterium. Other sponge species also harbor related Betaproteobacteria: the *Crambe crambe*‐associated bacterial community is dominated by a single betaproteobacterial symbiont (Croué et al., 2013) and several closely related betaproteobacterial OTUs are present in the sponge *Antho chartacea* (Taylor et al., 2007). Betaproteobacteria closely related to the latrunculid OTUs are also present in the cortex and endosome of *Tethya aurantium* (Thiel, Neulinger, Staufenberger, Schmaljohann, & Imhoff, 2007). All of these Betaproteobacteria form a sponge‐specific cluster and their phylogeny mirrors that of their hosts suggesting that these Betaproteobacteria are a novel clade of sponge symbionts that have coevolved with their hosts.

Sequence analysis (BLAST) of the 16S rRNA genes from the dominant Betaproteobacterial strains identified in this study placed these strains within the family Nitrosomonadaceae. Based on cultivation studies, bacteria within this family are lithoautotrophic ammonia oxidizers (Prosser, Head, & Stein, 2014). The oxidation of ammonia is an important component of nitrogen cycling in marine ecosystems where bioavailable nitrogen is often in limited supply (Fiore, Jarett, Olson, & Lesser, 2010; Moore et al., 2013). Acquisition of nitrogen by biota in these ecosystems typically occurs via recycling of nitrogen from organic matter or from nitrogen fixation, whereby N_2_ is reduced to NH_3_, and nitrification where ammonia is oxidized to nitrite or nitrate (Bayer, Schmitt, & Hentschel, 2008; Fiore et al., 2010). Nitrogen fixation and nitrification are processes carried out by prokaryotes. It has been established that several sponge species are able to excrete nitrate and/or nitrite suggesting the presence of nitrifying microbial symbionts within their mesohyls (Bayer et al., 2008; Diaz & Ward, 1997; Hoffmann et al., 2009; Jimenez & Ribes, 2007; Radax, Hoffmann, Rapp, Leininger, & Schleper, 2012). Indeed, several clades of nitrifying microbes have been recovered from sponges (Bayer et al., 2008; de Voogd et al., 2015; Hentschel, Piel, et al., 2012; Hoffmann et al., 2009; Karlińska‐Batres & Wörheide, 2013; Moitinho‐Silva et al., 2014; Radax et al., 2012; Taylor et al., 2007; Tian et al., 2016). While the presence of bacteria belonging to the clade Nitrosospira (family Nitrosomonadaceae) have been reported in sponges (Bayer et al., 2008; Karlińska‐Batres & Wörheide, 2013; Schmitt et al., 2012) and isolates from this clade have not been cultured to date, it is postulated that Nitrosospira are involved in nitrification in sponges (Bayer et al., 2008). The Nitrosomonadaceae strains in the Latrunculid sponges in our study were shown to be both numerically dominant as well as most likely metabolically active (Figure 7) suggesting that the symbionts in these sponges represent a source of bioavailable nitrogen for the sponge.

Several studies have reported the occurrence of Spirochaetae species within sponge microbiomes (Hentschel et al., 2002; Hentschel, Piel, et al., 2012; Neulinger, Stöhr, Thiel, Schmaljohann, & Imhoff, 2010; Taylor et al., 2007; Walmsley et al., 2012). Spirochaetae were found to be highly abundant in the calcareous sponge, *Clathrina clathrus*, comprising two morphotypes that are distributed evenly throughout the mesohyl. The 16S rRNA gene sequence of these two *C*. *clathrus* and four additional Spirochaetae OTUs from another *Clathrina* species share between 70.7% to 86.7% sequence identity indicating that all of these OTUs were distantly related to each other (Neulinger et al., 2010). The conservation of a single, dominant Spirochaetae OTU_(0.03)_ between sponge species within the same family has not been reported before. The distribution and relative abundance of the four closely related *Tsitsikamma* Spirochaetae OTUs appears to be genus‐specific, distinct from the less closely related S_OTU1, which occurs only in *C. bellae*.

What is the nature of the interactions between sponges and their dominant bacterial symbionts? The results of this and other studies provide evidence that the latrunculid Betaproteobacteria are members of a novel group of specialized symbionts that have coevolved with their sponge hosts. Data that support this proposal include: (1) their 16S rRNA gene sequences form a monophyletic cluster distinct (Group II, Figure 3) from those of Betaproteobacteria that are not dominant phylotypes in other sponge species and including several culturable sponge‐associated bacterial species (Group I, Figure 3); (2) the phylogenetic relationship between numerically dominant sponge‐associated Betaproteobacteria OTUs follows that of their host sponges; (3) FISH data suggest that Betaproteobacteria are intracellular in *Rhopaloeides odarabile* (Webster, Wilson, Blackall, & Hill, 2001) and we have preliminary data suggesting the same is true for *T. favus* (Waterworth, S. C., Dorrington, R. A., unpublished data); (4) the Betaproteobacteria are metabolically dominant in *T. favus* and *C*.* bellae* suggesting that sponge–bacterial interactions may involve the exchange of metabolites required for the survival of both organisms. Since related taxa are also found in other, non‐latrunculid sponges, from other geographical locations, the implication is that these dominant Betaproteobacteria symbionts may be a feature of the microbial communities associated with several other sponge families in addition to the Latrunculiidae.

The interaction between latrunculid sponges and Spirochaetae appears to be very different. Unlike the Betaproteobacteria, the presence of dominant Spirochaetae OTUs was confined to sponges belonging to the *Tsitsikamma* genus and C*. bellae* and they are not metabolically dominant. The *Tsitsikamma* and *C. bellae* Spirochaetae OTUs are phylogenetically closely related, but are only distantly related to other sponge‐associated Spirochaetae (<90%) implying that *Tsitsikamma* and *Cyclacanthia* sponges likely acquired these symbionts from their environment relatively recently, before they diverged from a common ancestor. Unlike other sponge‐associated spirochaetes, which belong to widely divergent taxa, the close phylogenetic relationship of the *Tsitsikamma* and *Cyclacanthia* spirochaetae suggest that they have a specialized function in the bacterial communities associated with these sponges. Sponges belonging to these two genera are known to produce tsitsikammamines and discorhabdins, the type and abundance of which are species dependent (Antunes et al., 2004; 2005; J. Kalinski & R. Dorrington, unpublished data). Given the distribution and relative abundance of the Spirochaetae OTUs within species from these two genera, it is tempting to speculate that these bacteria might be involved in pyrroloiminoquinone production.

In conclusion, this study has revealed the unprecedented conservation of two dominant bacterial symbionts within the Latrunculiidae family of marine sponges. Given the potent in vitro cytotoxicity of the pyrroloiminoquinone secondary metabolites that characterize the Latrunculiidae, this study raises interesting questions about the interactions between latrunculid sponges their dominant bacterial symbionts and the potential involvement of these bacteria in the biosynthesis of the pyrroloiminoquinones. A comparative study of the bacterial symbionts in latrunculid sponges species from other parts of the world, for example, New Zealand, which produce similar pyrroloiminoquinone metabolites, could potentially shed new light on the biosynthetic origin of these compounds.

## Conflicts of Interest

The authors declare no conflict of interest.
