# Supplementary figures and images for: Keeping it in the family: Coevolution of latrunculid sponges and their dominant bacterial symbionts
Source: Microbiologyopen. 2016 Oct 26;6(2):e00417. doi: 10.1002/mbo3.417 (PMC5387304; doi:10.1002/mbo3.417)

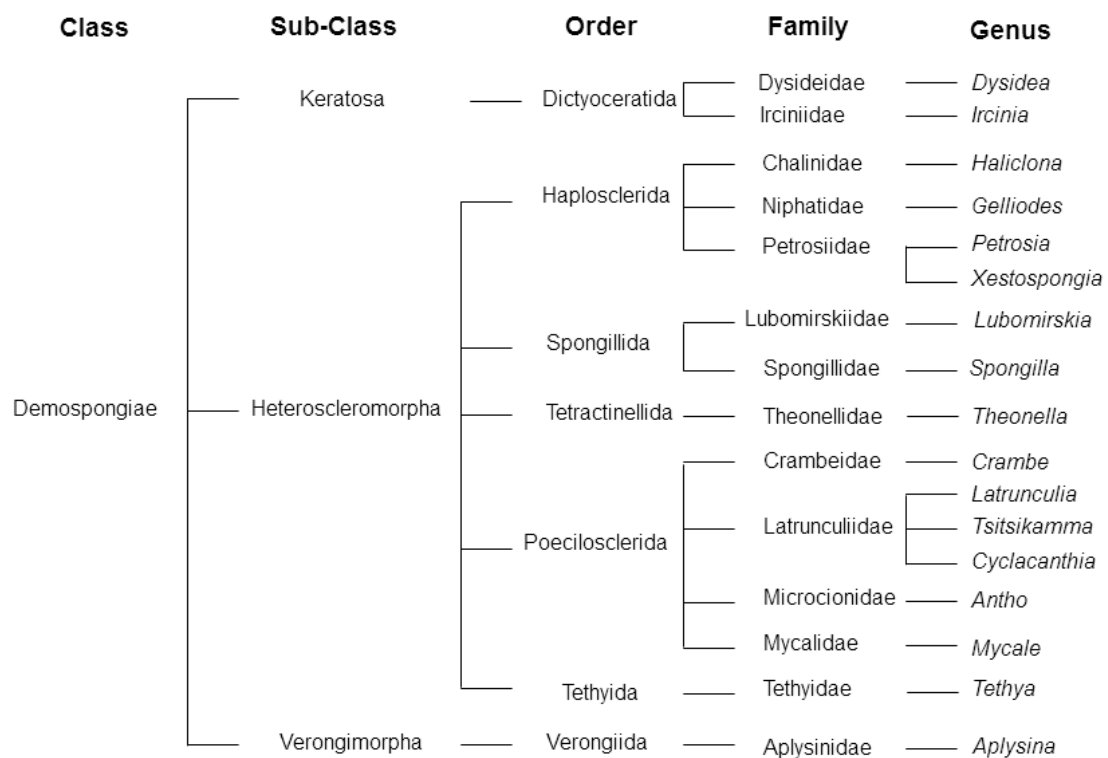

Supplementary Figure 1 : Taxonomic relationship between sponge species relevant to this study.

Supplement: Supplementary file 1 [file MBO3-6-na-s001.pdf]
