# Supplementary material for: Keeping it in the family: Coevolution of latrunculid sponges and their dominant bacterial symbionts
Source: Microbiologyopen. 2016 Oct 26;6(2):e00417. doi: 10.1002/mbo3.417 (PMC5387304; doi:10.1002/mbo3.417)
Supplement: Supplementary file 2 [file MBO3-6-na-s002.docx]

**Table S1**: Summary of 16S rRNA amplicon pyrosequencing data used in this study

| **Collection Number** | **Taxonomic**  **identification** | **Number of reads in the data set^1^** | **Number of OTUs at a distance of 0.0** | **Number of OTUs at a distance of 0.03** |
| --- | --- | --- | --- | --- |
| TIC2009-002 | *Tsitsikamma* sp. 001 | 891 | 435 | 71 |
| TIC2010-070 | *Tsitsikamma* sp. 005 | 14070 | 3081 | 333 |
| TIC2010-2B | *Tsitsikamma* sp. 004 | 4497 | 1916 | 306 |
| TIC2010-031 | *Latrunculia algoaensis* | 10460 | 1808 | 265 |
| TIC2010-030 | *Mycale (Mycale)* sp. 001 | 22625 | 3461 | 378 |
| TIC2011-102 | *Tsitsikamma* sp. 002 | 11217 | 2700 | 327 |
| TIC2012-057 | *Tsitsikamma* sp. 001 | 3260  2960 (gDNA)  2918 (cDNA) | 980  1174  1224 | 365  306  312 |
| TIC2012-056 | *Cyclacanthia* *bellae* | 2959  2061(gDNA)  2040 (cDNA) | 947  1336  862 | 285  378  192 |
| TIC2014-001 | *Tsitsikamma* sp. 001 | 17706 | 3516 | 830 |
| Sediment | NA | 3281 | 2539 | 1068 |
| Seawater | NA | 2218 | 1313 | 337 |

^1^ Number of sequences remaining in the data set subsequent to data curation which included removal of chimeras, short sequence reads, reads with ambiguities, as well as reads which could not be classified as bacterial in origin.
